# Methicillin resistant *Staphylococcus aureus *adhesion to human umbilical vein endothelial cells demonstrates wall shear stress dependent behaviour

**DOI:** 10.1186/1475-925X-10-20

**Published:** 2011-03-22

**Authors:** Kayla D Viegas, Sharul S Dol, M Mehdi Salek, Robert D Shepherd, Robert M Martinuzzi, Kristina D Rinker

**Affiliations:** 1 Department of Mechanical Engineering, Schulich School of Engineering, University of Calgary, Calgary, AB, Canada; 2 Department of Chemical and Petroleum Engineering, Schulich School of Engineering, University of Calgary, Calgary, AB, Canada; 3 Centre for Bioengineering Research and Education, Schulich School of Engineering, University of Calgary, Calgary, AB, Canada; 4 Department of Physiology and Pharmacology, Faculty of Medicine, University of Calgary, Calgary, AB, Canada; 5 Calvin, Phoebe and Joan Snyder Institute of Infection, Immunity and Inflammation, Faculty of Medicine, University of Calgary, Calgary, AB, Canada

## Abstract

**Background:**

Methicillin-resistant *Staphylococcus aureus *(MRSA) is an increasingly prevalent pathogen capable of causing severe vascular infections. The goal of this work was to investigate the role of shear stress in early adhesion events.

**Methods:**

Human umbilical vein endothelial cells (HUVEC) were exposed to MRSA for 15-60 minutes and shear stresses of 0-1.2 Pa in a parallel plate flow chamber system. Confocal microscopy stacks were captured and analyzed to assess the number of MRSA. Flow chamber parameters were validated using micro-particle image velocimetry (PIV) and computational fluid dynamics modelling (CFD).

**Results:**

Under static conditions, MRSA adhered to, and were internalized by, more than 80% of HUVEC at 15 minutes, and almost 100% of the cells at 1 hour. At 30 minutes, there was no change in the percent HUVEC infected between static and low flow (0.24 Pa), but a 15% decrease was seen at 1.2 Pa. The average number of MRSA per HUVEC decreased 22% between static and 0.24 Pa, and 37% between 0.24 Pa and 1.2 Pa. However, when corrected for changes in bacterial concentration near the surface due to flow, bacteria per area was shown to increase at 0.24 Pa compared to static, with a subsequent decline at 1.2 Pa.

**Conclusions:**

This study demonstrates that MRSA adhesion to endothelial cells is strongly influenced by flow conditions and time, and that MSRA adhere in greater numbers to regions of low shear stress. These areas are common in arterial bifurcations, locations also susceptible to generation of atherosclerosis.

## Background

Infections of the cardiovascular system, including those involving prostheses and devices, are a globally recurring problem. Vascular infections are often life-threatening, spread easily, and costly to treat. Furthermore, infection is a common problem affecting the success of biomedical implants, such as vascular stents [1]. Bacteria can be introduced through surgical interventions, travel through the bloodstream and infect the endothelial cells lining the blood vessels. Cardiovascular disease has also been linked to microbial infection [2,3], with attachment of bacterial pathogens to endothelium or extracellular matrix being an initial step in the process [4].

*Staphylococcus aureus *is a highly adaptable microbial pathogen that is considered to be a leading cause of various community- and hospital-acquired infections [5]. The frequency of *S. aureus *infection has risen dramatically over the last few decades in accordance with the number of patients receiving vascular implants, such that *S. aureus *accounts for the majority of device-related infections [6]. In addition, *S. aureus *is the primary cause of endovascular and endocardial infections that are extremely difficult to treat, with mortality rates between 40 and 50% at one year [7]. Of particular concern is the ability of *S. aureus *to become tolerant to antibiotic therapies, and to generate antibiotic resistant strains.

One such strain is Methicillin resistant *Staphylococcus aureus *(MRSA). In addition to its intrinsic resistance to methicillin, MRSA is also insensitive to other common antibiotics such as oxacillin, penicillin and amoxicillin [8]. MRSA was first identified in 1961 and is now the most common antibiotic-resistant bacteria worldwide [8]. MRSA infection rates have continued to grow in recent decades: in 1974, 2% of *S. aureus *infections in US intensive-care units were caused by MRSA, in 1995 this number was 22%, and in 2003, 64% [9]. Approximately 94,000 Americans suffer each year from invasive MRSA infections, and an estimated 19,000 of these incidences (20%) result in death [10]. Globally, MRSA is known to be associated with longer hospital stays, increased mortality, morbidity and much higher costs of treatment.

The location, strength, and magnitude of vascular bacterial adhesion, along with subsequent cellular interactions involved in disease progression, are likely to be highly dependent on the hydrodynamic environments experienced within affected vessels. Once in the bloodstream, bacteria contact the vascular endothelium. Endothelial cells mediate many vascular functions including inflammatory responses and transendothelial migration of nutrients, biological molecules, and leukocytes into the surrounding tissue [11]. These functions occur under the dynamic conditions of blood flow, and are strongly affected by hemodynamic forces. Shear stress levels ≥1 Pa, which generally occur through straight portions of the arteries, typically correspond to healthy, disease resistant regions of the vasculature, while low shear stress (≤ 0.4 Pa), which is found at arterial branch points and areas of curvature, often corresponds to areas susceptible to both atherosclerosis and microbial adhesion [12-14]. Receptor-ligand adhesive bonds are strongly affected by flow conditions. While it would seem from intuition and theory that receptor-ligand bonds should form less frequently and break more quickly under flow, experimental evidence has shown that in some cases the opposite occurs. We previously showed monocyte adhesion to HUVEC to be shear stress dependent, with stronger adhesion occurring with increased shear stress [15], possibly due to increased cell or microvilli deformation [16]. However, the bonds themselves may respond differently depending on the level of force (from shear stress, atomic force microscopy, or other methods). Adhesive bonds are characterized as 'slip' bonds if bond lifetimes decrease under increased force and catch bonds if the opposite occurs. While many studies have focused on P- and L-selectins involved in leukocyte adhesion [17,18], a catch bond has been identified in *Escherichia coli *[19,20]. Therefore it is essential to observe adhesive interactions under physiologically relevant conditions. This may enable establishment of more accurate models of vascular infection in the human body and evaluation of specific adhesion mechanisms. A few studies have investigated *S. aureus *adhesion to endothelium under flow [21-23], however none of these involve MRSA.

*In vitro *systems have been developed that allow cultured endothelial cells to be exposed to well-defined flow conditions, and include orbital shakers [24], cone and plate viscometers [25,26], and parallel-plate flow chambers (PPFC) [15,16,27-31]. PPFC enable establishment of fully developed laminar flow within the channel of the chamber and theoretically should allow exposure of cells to uniform flow fields. However, many PPFCs have been found to not provide uniform shear stresses across the surface of the chamber [32-34]. Therefore, cell responses may not be consistent over the chamber area [34].

This study investigated the hypothesis that shear stress inhibits MRSA adhesion and subsequent infection of vascular endothelium. The objectives of this study were to first evaluate the performance of a newly designed PPFC for its ability to provide defined flow and uniform shear stress to cultured cells, and second, to use this PPFC to analyze the influence of shear stress on MRSA infection of endothelium. Human umbilical vein endothelial cells (HUVEC), were selected since they represent a good endothelial model and are widely used in the field [15,16,31]. Further, since many *S. aureus *experiments have been performed with HUVEC or the HUVEC cell line EA hy926, this enables closer comparisons with previous data [22,23,35-37].

## Methods

### Bacterial culture

The bacterial strain used for all experiments, MRSA UC18, a hospital-acquired isolate, was kindly provided by Dr. Howard Ceri (University of Calgary). MRSA was routinely cultured in Tryptic Soy Broth (TSB) at 37°C and 100 rpm. Bacterial growth was quantified using optical density (OD) at a spectrophotometer light wavelength of 600 nm and viable plate counts. Cells passaged from actively growing cultures were maintained at 37°C under constant rotation, and harvested at an OD of 0.50, after ~3 h of incubation. As determined by growth curves, this point corresponded to mid-exponential phase. All bacterial ODs were correlated to colony counts (measured in colony forming units; CFU). Prior to adhesion studies, bacteria were labelled for 30 minutes at 37°C with a 1:1000 dilution of SYTO 9 (Invitrogen, Grand Island, NY, USA) in Dulbecco's Modified Eagle's Medium (DMEM, Sigma-Aldrich, St. Louis, MO, USA) supplemented with 2% Fetal Bovine Serum (FBS). After labelling, cells were washed twice with TBS and diluted in DMEM to a concentration of 10^7 ^CFU/mL.

### Endothelial cell culture

Pooled Human Umbilical Vein Endothelial Cells (HUVEC; Lonza, Walkersville, MD, USA) were cultured from supplied stock and expanded up to a maximum of passage 6. Tissue culture flasks and glass slides were coated with 0.1% gelatin (Difco, Becton, Dickinson, Sparks, MD, USA) in M199 (Sigma-Aldrich) prior to cell seeding. Slides were usually confluent and ready for use after 2 days.

### In vitro flow model

**Figure 1:**
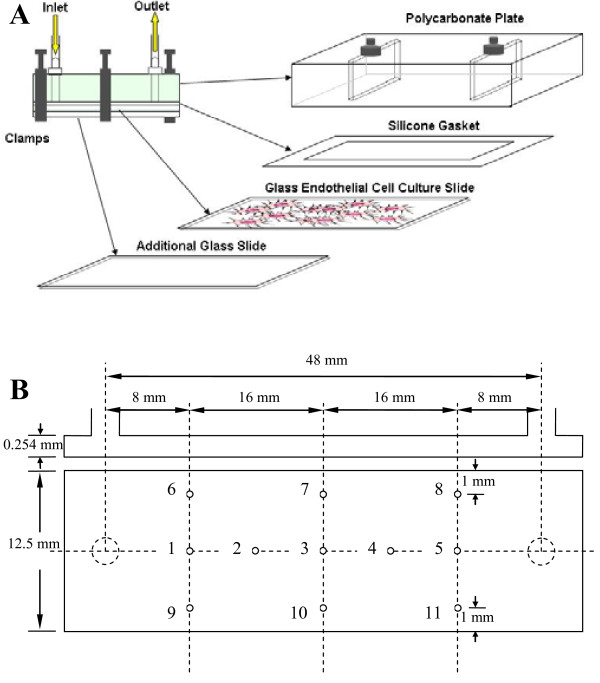
**Experimental setup**. (A) Schematic of the flow chamber; (B) Flow chamber nomenclature and PIV measurement locations.

The PPFC system and methods used for this study were similar to that used by Shepherd et al., 2009 [30]. As shown in Figure 1A, the flow chamber was comprised of a polycarbonate top plate with ports for an inlet and outlet, a 254 μm thick silicon rubber gasket (Specialty Manufacturing, Saginaw, MI, USA) with a removed rectangular section in the center to form the flow channel (clear section: *w *= 1.25 cm, *l *= 4.6 cm) and a glass microscope slide with a confluent monolayer of HUVEC. DMEM containing labelled MRSA was pumped using a variable speed peristaltic pump (MasterFlex^® ^L/S^®^; Cole Parmer, Montreal, QC, Canada) through MasterFlex 16 Norprene Tubing from a media bottle, through a pulse dampener (Cole Parmer), to the flow chamber, and back into the media bottle. A heat lamp was used to maintain the temperature of the flow circuit at approximately 37°C. At the end of the experiments, slides were washed twice with HEPES Buffered Saline Solution (HBSS), fixed with 4% paraformaldehyde for 20 min, rinsed twice with PBS and mounted with VECTASHIELD^® ^Mounting Medium (Vector Labs, Burlington, ON, Canada) and a cover slip. The nominal average wall shear stress, τ_w_, for flow in the channel was determined by:

where *Q *is the volumetric flow rate (2 ml min^-1 ^or 10 ml min^-1^), *μ *is the fluid viscosity, *w *is the channel width (12.5 mm), and *h *is the flow path height. For this chamber, the width to height ratio was 49. For a Newtonian fluid, shear rate, , is related to shear stress according to:

For each flow condition investigated, the Reynolds number was calculated in order to verify that flow within the channel was laminar. The following formula was used:

According to the PIV analysis (see below), the height of the HUVEC and extracellular matrix layer was 20.1 ± 3.9 μm [38]. This height was subtracted from the channel height (254 μm ) to determine the height of the flow path, h. The density and viscosity of the culture media were 0.9852 g·cm^-3 ^and 0.831 cP (determined using a capillary viscometer (Cannon-Fenske; Cannon Instrument Company State College, PA, USA), respectively. These values yield shear stresses of 0.24 Pa and 1.2 Pa (at shear rates of 293s^-1 ^and 1460s^-1 ^and Reynolds numbers of 6.57 and 32.9, respectively) for the conditions used in the infection study. The Reynolds numbers less than 1200 indicate that flow is in the laminar regime. Physiological values of Reynolds numbers (200-6000) could not be reproduced due to the high flow rates needed [39].

The infection data was normalized to separate adhesion kinetics from transport effects by the method of Munn et al. [15,40,41]. The settling velocity was calculated as:

where ρ is the density of the fluid, ρ_c _is the density of the bacterium (1.45 g cm^-3 ^[42]), g is gravitational force, r is the radius of the bacterium (0.5 μm) and μ is the viscosity of the medium. The dimensionless concentration in the steady state region of the flow chamber is determined as follows:

where *ψ *is the steady state concentration near the surface, *C *is the concentration of bacteria in the bulk fluid (1 × 10^7 ^cells ml^-1^), *x *is the distance from the inlet (2.3 cm), *u *is the velocity and *h' *is the height of fluid (taken to be one cell diameter, 1 μm [41,43]). The velocity, *u*, was determined using the method of Goldman et al. [44] to take into account the lower velocity of a neutrally buoyant sphere near the wall. The dimensionless concentration was normalized against the value determined for the static condition then multiplied by the experimentally determined values as in previous work [15].

The forces acting on a bacterium attached to an endothelial cell were determined using the method discussed in Rinker et al. [15]. The drag force, F_x_, is given by

where *r *is the cell diameter (1 μm) and t is the shear stress. F_x_* is equal to 1.7005 for the case where the bacterium is touching the surface [44].

### Imaging

An Olympus Fluoview FV1000 Confocal Scanning Laser Microscope (CSLM) was used to take fluorescent image stacks using a water-immersed, coverslip-corrected 60× objective and an Argon laser. For each condition, a minimum of three replicates were analyzed. For each experiment, between 4 and 12 confocal stacks (thickness of slice = 0.5 μm) were taken randomly along the flow channel. This generated data encompassing adhesion to a minimum of 100 HUVEC per experiment. Within each image stack taken, all fully visible HUVEC were analyzed, with any cell only partially contained in the field of view excluded. All HUVEC with one or more adherent MRSA were considered infected. The number of HUVEC associated MRSA were counted on a per-cell basis. The area analyzed in each picture was 0.001 cm^-2^.

### PIV measurements

Flow field information was obtained for the parallel plate flow chamber with a micro-PIV system (TSI) as previously reported [38]. Figure 1B shows the measurement locations inside the chamber. Duke red fluorescent particles (2.0 μm nominal diameter) were used as tracers (concentration of 0.3% by volume). The particles were illuminated with a 532 nm Nd:YAG laser. A Nikon Plan Fluor 10X 0.3 NA objective lens was used with an inverted Nikon microscope. The images were captured by a CCD camera (POWERVIEW 4M; 2048 × 2048 pixels) with a 2× projection lens giving an effective view field of 760 μm × 760 μm. A volume illumination technique was used to define the PIV measurement plane [45]. The velocity field was estimated from ensemble averages of 100 image pairs acquired at a rate of 7.5 Hz with an inter-image time delay of 300 μs to 500 μs. TSI Insight^® ^software was used to analyze the captured images using a cross-correlation technique with a Gaussian window function. The spatial resolution, based on 64 × 64 pixel interrogation spots, was 11.84 μm/spot. A standard 50% interrogation area overlap was used to represent the vector field. The local shear stress was estimated from the velocity gradient at the wall:

The velocity gradient was estimated using a three-point fit. The overall measurement uncertainty is estimated to be ± 2% and ± 6% for the local velocity and wall shear stress, respectively, based on pixel resolution and repeatability.

The uncertainty in the location is related to the PIV measurement depth, *δz*, which is related to the depth of focus of the recording lens and can be estimated by considering the effects of: diffraction, geometric optics and the finite size of the particle [45]:

where n is the refractive index of the fluid between the micro-fluidic device and the objective lens; λ_0 _is the wavelength of light, in vacuum, imaged by the optical system; *NA *is the numerical aperture of the objective lens; *θ *is the light collection angle and *d_p _*is the particle diameter. The resulting measurement depth uncertainty is about 20 μm.

### CFD modeling

**Figure 2:**
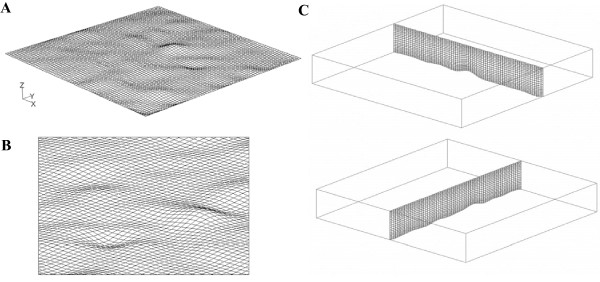
**Computational grids for the endothelial cell surfaces**. Determined at (A) the full range and (B) the zoomed-in surfaces; (C) at two perpendicular planes (X-Z and Y-Z).

FLUENT version 6.3.2 was used to model the flow through the PPFC in the absence (clean chamber) or presence of cells using grids generated in Gambit. The steady three-dimensional forms of the continuity and Navier-Stokes equations were solved with constant media properties at 20°C. For the clean flow chamber case, a structured, rectangular grid was used. For the case with endothelial cells, the endothelial cell surfaces were reconstructed in Gambit using elevations measured from micro-PIV at 225 points in a plane of 700 *μ*m × 700 *μ*m. The velocity profile was measured using micro-PIV at each of these 225 points to locate the surface (according to no slip boundary condition at the solid surfaces). Interpolation was used to construct the surface based on these 225 points. In this method the interpolated surface was constrained to pass through all measured vertices with zero tolerance, while minimizing the curvature. Hexagonal grids with low skewness (< 0.4) were generated in the domain. Computational grids for the endothelial cells surfaces and two perpendicular planes are shown in Figure 2. A grid sensitivity analysis was conducted for different node-density grids to select the optimum grid (the optimum grid included 98000 computational cells in this study). A double-precision solver was used which is more accurate for the geometries with very disparate length scales. The momentum equations were solved with first order upwind schemes and the SIMPLE algorithm was used for the pressure velocity coupling.

For the PPFC without cells, a uniform velocity profile was used at the inlet and a no slip boundary condition was applied at the walls. Outflow boundary condition was used at the outlet: the flow variable gradients normal to the boundary are set to zero. For the PPFC with endothelial cells, the inlet velocity profile measured experimentally was used at the beginning of the solution domain. No slip boundary conditions were applied at the lateral walls and endothelial cell surfaces. Additional details of the CFD analysis are provided in Dol et al. [38].

### Statistical analysis

Data is reported as the mean +/- the standard error of the mean (SEM). Systat (v. 13, Systat Software Inc, Chicago, IL) was used to perform analysis of variance (ANOVA). A Tukey post-test analysis was performed on data pairs. P-values < 0.05 (corresponding to a 95% confidence interval) were considered statistically significant.

## Results

### MRSA adhered to HUVEC and was internalized

**Figure 3:**
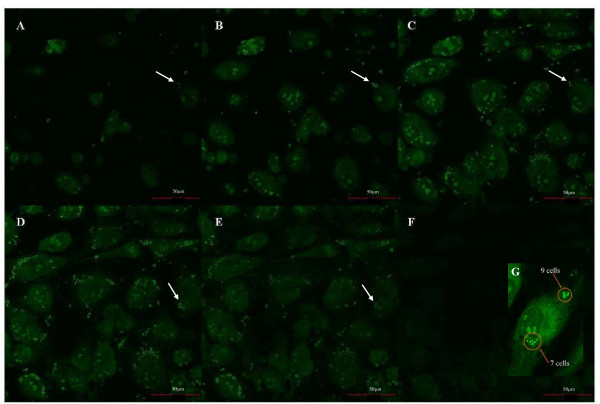
**MRSA adhesion and infection of endothelial cells**. Selected confocal image slices (0.5 μm thick) from one stack (from (A) the top of the cells through to (F) the bottom of the cells) were taken of HUVEC infected with MRSA under static conditions for 30 minutes. The brighter green dots are adherent MRSA UC18 cells. The arrows point to the same cell in each slice. In (A) only 2 MRSA can be seen at this location, whereas there is a cluster of 7 at the same location in the next slice (B). Two MRSA are seen at the same location in (C). Intracellular MRSA are more easily seen for this cell in (D). The inset in (F) shows a magnification of one cell and identification of bacteria (G). Since there is more than one adherent MRSA, this cell would be considered to be infected. If only this slice was used to analyze this particular endothelial cell, 16 MRSA would be used to determine the average number of MRSA per HUVEC. However, the entire stack was analyzed as cells may be bound in different planes along the height of the cell.

**Figure 4:**
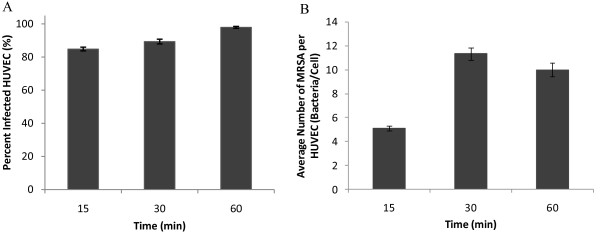
**MRSA infection of HUVEC under static conditions**. (A) Percentage of infected HUVEC and (B) number of MRSA per HUVEC versus time. HUVEC were exposed to SYTO-9 labelled MRSA for 15, 30, or 60 minutes then analyzed by confocal microscopy to quantify the number of MRSA per HUVEC cell as described in Figure 2. Number of samples analyzed: 15 min: 3 experiments, 556 HUVEC, 2790 MRSA; 30 min: 3 experiments, 411 HUVEC, 4495 MRSA; 60 min: 3 experiments: 423 HUVEC, 4078 MRSA. Error bars are plotted as standard error of the mean (SEM).

In order to assess binding of MRSA to endothelium, experiments were first performed under static conditions to develop a method to quantify infection and to determine time dependence. HUVEC were exposed to MRSA collected during exponential growth, during which bacteria have a higher capacity to adhere to and infect host cells [46-48]. After 30 minutes of adhesion under static conditions, MRSA were seen in multiple slices of the image stack (Figure 3). MRSA were found on the surface and in the cytoplasm of the HUVEC. The distribution of bacteria deviated greatly from cell to cell with some endothelial cells having none to very few bound bacteria, with others having many bound bacterial cells. At time points of 15, 30, and 60 minutes, the maximum number of MRSA attached to or inside a single HUVEC were observed to be 31, 68, and 75 respectively (data not shown). MRSA UC18 is a virulent *S. aureus *strain. 85% of the exposed HUVEC were infected after 15 minutes and 98% after 60 minutes; an increase of 15% (p < 0.001; Figure 4A). The average number of MRSA per endothelial cell increased 122% between time points of 15 and 30 minutes (p < 0.001; Figure 4B). However, from 30 minutes to 60 minutes, this number did not change statistically.

### Flow chamber provided uniform flow field

**Figure 5:**
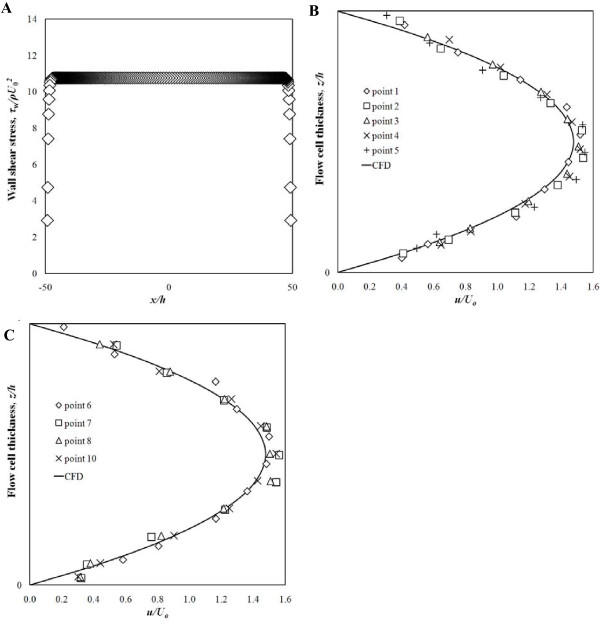
**Streamwise velocity profile of flow in the parallel-plate flow chamber determined using micro-PIV and CFD**. Streamwise velocity characterization (A) along the center and (B) in proximity of the side walls. (C) Shear stress distribution along the channel span at *z*/*h *= 0, 1. (B) A peak at coordinates (*x*, *y*) = (250 *μ*m, 650 *μ*m) and (C) a trough at coordinates (*x*, *y*) = (300 *μ*m, 350 *μ*m).

Next, we wanted to assess the role of fluid shear stress on MRSA adhesion. A parallel plate flow chamber was designed, fabricated and tested. This device was found to provide uniform flow over 95% of the flow surface, with flow field variation only observed in the vicinity of the lateral walls. A representative cross-section is shown in Figure 5A. All 11 measurement points met target shear stress levels indicating that the influence of the inlet and outlet ports on the flow in the test section was not significant. Characteristic laminar parabolic velocity profiles were observed at locations along the centreline and near the lateral walls (Figures 5B and 5C). Fluid shear stress in the chamber determined by PIV agreed with CFD results within experimental uncertainty for two target shear stress levels (0.200 ± 0.006 Pa and 1.00 ± 0.06 Pa).

### Endothelial cells increased shear variations

**Figure 6:**
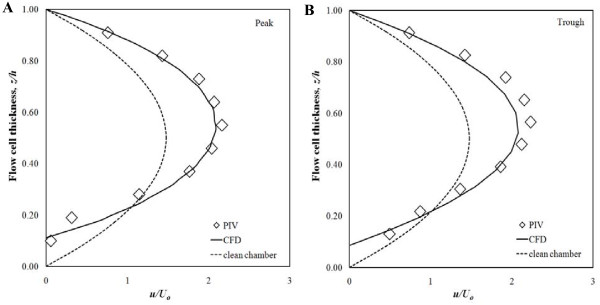
**Local distortion of the streamwise velocity profile due to endothelial cell topography**. Velocity profiles were determined at point 6 from Figure 1B; (A) peak (*x*, *y*) = (250 *μ*m, 650 *μ*m); (B) trough (*x*, *y*) = (300 *μ*m, 350 *μ*m).

**Figure 7:**
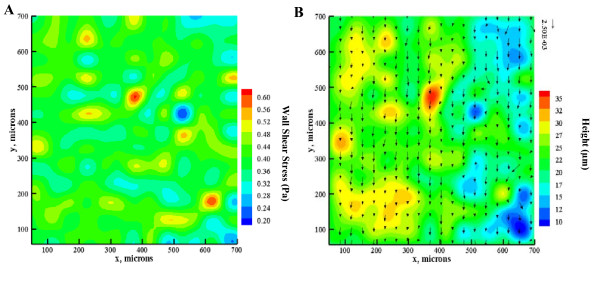
**Flow field and surface height changes due to presence of endothelial cells**. Velocity field and topography at point 6. (A) Contour of wall shear stress (unit is in Pa) over the endothelial cells as obtained by CFD; (B) PIV velocity field over the contour of cell elevation (unit is in microns).

Further testing of the flow chamber was performed with PIV and CFD to determine the effect of the layer of endothelial cells on fluid shear stress levels. Changes in the channel surface topography due to the presence of the endothelial cells induced local variations in the velocity profiles (Figure 6) and, consequently, the surface shear stress experienced by the cells. In the peak regions, the near surface flow speeds were larger than in the trough regions, resulting in a higher shear at the peaks than in the troughs (Figure 7A). The spatial variations in the surface shear stress distribution can be attributed directly to local changes in the cell-matrix thickness (Figure 7B). It was found that the no-slip condition remained valid and that the predicted and measured local surface shear stresses agreed within experimental uncertainty.

### Effect of shear stress on MRSA infection of HUVEC

**Figure 8:**
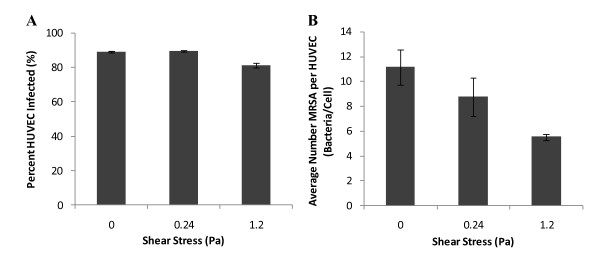
**Shear stress dependence of MRSA infection of HUVEC**. (A) Percentage of infected HUVEC and (B) number of MRSA per HUVEC versus magnitude of shear stress exposure during infection for 30 minutes. Number of samples analyzed: Static: 3 experiments, 411 HUVEC, 4495 MRSA; 0.24 Pa: 4 experiments, 462 HUVEC, 3795 MRSA; 1.2 Pa: 4 experiments: 546 HUVEC, 3000 MRSA. Error bars are plotted as standard error of the mean (SEM). All conditions in part (B) are significant to a level of p < 0.001. In part (A), the percent infected HUVEC at 1.2 Pa was significantly different than that at 0.24 Pa or static conditions to a level of p < 0.001.

The characterized PPFC was then used to evaluate adhesion of MRSA to HUVEC under flow. A 30 minute exposure time was selected since this time point yielded significant adhesion under static conditions, but was short enough that major physiological changes for endothelium and MRSA were less likely to be occurring. Further (as discussed previously), there was no significant increase in adhesion after this point in static studies. When HUVEC were exposed to MRSA in flowing media, the percentage of infected HUVEC decreased 9.3% between 0.24 Pa and 1.2 Pa (p < 0.001; Figure 8A). However no significant difference was found between static and 0.24 Pa. The average number of MRSA per HUVEC was found to decrease with increasing shear stress (Figure 8B) with a 22% decrease from static to 0.24 Pa (p < 0.001) and a further 37% decrease between 0.24 Pa and 1.2 Pa (p < 0.001). The drag force on the attached bacterium was calculated for each flow condition. Adherent MRSA were exposed to a maximum drag force of 1.92 pN at 0.24 Pa and 9.61 pN at 1.2 Pa.

### MRSA infection under flow varied with time

**Figure 9:**
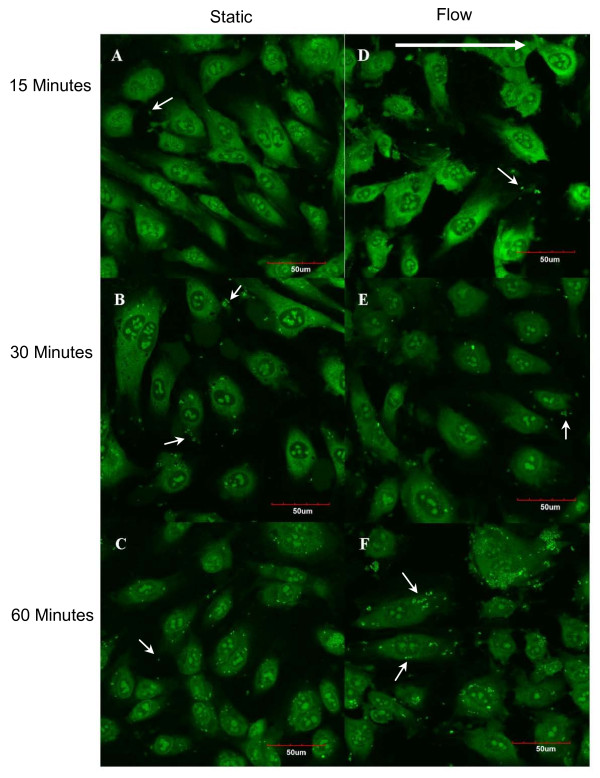
**MRSA infection of HUVEC under flow**. Confocal image slices taken after MRSA infection of HUVEC under static (A-C) or flow conditions at 0.24 Pa (D-F) for infection times of 15 minutes (A, D), 30 minutes (B, E), and 60 minutes (C, F). The large arrow indicates the direction of flow. The small arrows show representative bacteria. Bar represents 50 μm. MRSA labelled with SYTO-9.

**Figure 10:**
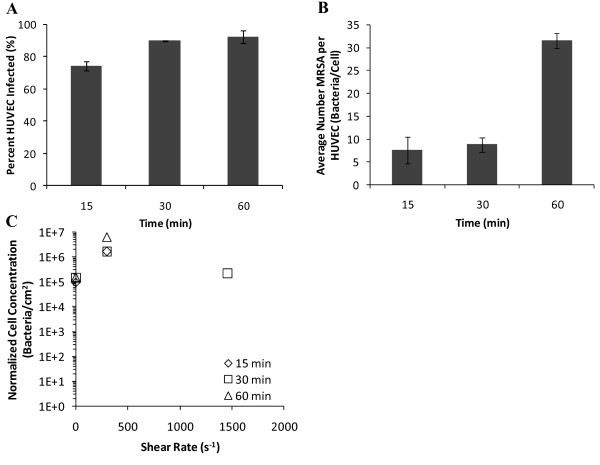
**MRSA infection of HUVEC under flow versus time**. (A) Percentage of HUVEC infected and (B) average number of adherent or internalized MRSA per HUVEC for dynamic MRSA adhesion experiments versus time at 0.24 Pa. (C) The number of experimentally determined bacteria per area normalized against the dimensionless cell concentration versus shear rate. The error bars for SEM were smaller than the markers for each data point and were not included in the graph. Number of samples analyzed: for static conditions: 15 min: 3 experiments, 556 HUVEC, 2790 MRSA; 30 min: 3 experiments, 411 HUVEC, 4495 MRSA; 60 min: 3 experiments: 423 HUVEC, 4078 MRSA. For 293s^-1^: 15 min - 3 experiments, 519 HUVEC, 3959 MRSA; 30 min- 4 experiments, 462 HUVEC, 3795 MRSA; 60 min- 3 experiments: 523 HUVEC, 14,932 MRSA; For 1460s^-1 ^at 30 min: 4 experiments, 546 HUVEC, 3000 MRSA. Error bars are plotted as standard error of the mean (SEM).

Additional experiments were performed to determine if MRSA adhesion and internalization under flow followed the same trend with time as the experiments performed under static conditions. A low shear stress case (shear stress 0.24 Pa; shear rate 293 s^-1^) was selected to determine if saturation of adhesion sites was occurring in adhesion of MRSA to endothelium. MRSA adhered to HUVEC from the start of incubation through 60 minutes, at which time a clear difference was seen between static and dynamic MRSA adhesion and internalization (Figure 9). Analysis of the confocal stacks revealed the majority of MRSA appeared to be distributed along the periphery of the endothelial cells, with few cells attached to the region above the nucleus under flow. For the static condition, it was difficult to find bacteria at the 15 minute time point. However, bacteria are readily seen at 30 and 60 minutes. Under flow, there is a dramatic increase in the number of bacteria between 30 and 60 minutes. As shown in Figure 10A, the percent of HUVEC infected by MRSA under flow was 12% lower at 0.24 Pa compared to static conditions after 15 minutes of infection (74.4% vs 84.8%; p < 0.03). At 0.24 Pa, the percent of infected HUVEC increased 20% between 15 and 30 minutes (p < 0.001) with no significant change between 30 and 60 minutes (Figure 10A). There was no significant change in the number of MRSA per HUVEC between 15 and 30 minutes; however, between 30 minutes and 60 minutes there was a 260% increase in the number of bacteria (p < 0.001; Figure 10B). This was a dramatic increase; a trend which differed from the time course results found under static conditions.

In order to correct for differences in bacterial concentration near the endothelial surface with increasing shear stress, the dimensionless cell concentration was determined and used to normalize the data. The number of bacteria per area was determined for each condition. This data had similar trends to the number of MRSA per HUVEC data already presented. When the experimentally determined bacteria per area was corrected for the dimensionless cell concentration near the surface, there was an increase in MRSA per area from static conditions to a shear rate of 293s^-1 ^(0.24 Pa) and a decrease in MRSA per area at 1460s^-1 ^(1.2 Pa) at the time point of 30 minutes (Figure 10C).

## Discussion

This study demonstrated that MRSA adheres to human endothelial cells in a manner dependent on fluid flow. The highest adhesion was found at the low shear stress condition (0.24Pa), which is a level found in arterial bifurcations. As fluid forces increased to levels seen in straight portions of arterial vessels, normalized MRSA adhesion decreased. Results also showed the capabilities of micro-PIV and CFD in determining the flow field over endothelial cells and the wall shear stress distribution over irregular cell surfaces.

Through analysis of image stacks obtained from confocal microscopy, MRSA infection of HUVEC was found to be heterogeneous (with some HUVEC experiencing a high degree of infection compared to others) and localized. Internalized bacteria were found as early as 15 minutes. For all conditions, most of the adherent bacteria were found around the periphery of the HUVEC. Becker *et al. *[49] made the same observation and hypothesized that this could be attributed to the fact that these pericellular regions are rich in fibronectin. Based upon our numerical simulations, PIV results and adhesion assays, we predict that there are also micro-scale shear stress effects at play during adhesion under fluid flow. The shear stress over the surface of each endothelial cell reaches a minimum in the junctional areas and a maximum above the cell nucleus. This leads to fluid velocity variations near the monolayer surface that impact cell trajectories. These factors may explain why we observed an increase in MRSA adherence in the area between adjacent cells.

Before normalization, our results showed that the number of MRSA per HUVEC decreased after 30 minutes of steady flow at 293 s^-1 ^(0.24 Pa) as compared to behaviour under static conditions, and decreased further with increasing shear rate and stress (1.2 Pa; 1460s^-1^). These results are similar to those of Shenkman et al. [23] who found a 2.5 fold decrease in *S. aureus *8325-4 adhesion to a HUVEC cell line after 20 minutes at a shear rate of 200s^-1 ^compared to static conditions. The larger fold decrease found by Shenkman et al. may be due to strain-specific differences in adhesion. The same group found no change in *S. aureus *RN6390 adhesion to endothelium after 20 minutes at a shear rate of 200s^-1 ^compared to static [22]; indicating a dependence on strain of *S. aureus*. Further, Reddy and Ross found no adhesion for *S. aureus *8325-4 to Bovine Aortic Endothelial Cells for any shear rate (1s^-1^- 200s^-1^) [21]; indicating a possible dependence on type and species of endothelial cell. However, there may be other factors contributing to the different results seen by Shenkman et al. and Reddy and Ross [23,21]. The growth phase for maximal adherence is strain specific [47]. Reddy and Ross used bacteria in the early exponential phase while Shenkman et al. used bacteria in the stationary phase. At later growth phases, *S. aureus *may increase levels of an adhesin important for adhesion under flow. Further, Reddy and Ross counted surface bound bacteria, whereas Shenkman et al. measured radioactivity and thus intracellular as well as attached bacteria. In our work, we analyzed stacks obtained from confocal microscopy to quantify intracellular and extracellular bacteria, which may explain why our results agree more closely with Shenkman et al. [23].

When our results are corrected for differences in cell flux to the endothelial surface under flow, bacteria per area was shown to increase with low levels of shear (293 s^-1^; 0.24 Pa), then decrease at the higher shear condition (1460 s^-1^; 1.2 Pa). Lower levels of adhesion may be observed at high shear rates because adhesin-receptor bonds are less likely to form at the shortened contact times [15]. A similar trend was seen by Boks et al (2008) for *Staphylococcus epidermidis *on glass [50]. An analysis of the drag forces on the attached bacteria shows values of 1.9 pN to 9.6 pN for the conditions of this study. This force was calculated for a bacterium attached to the highest point on the endothelial cell and therefore represents a maximum. Lower forces would be present in the valleys between the cells as discussed previously. The force required to detach *S. aureus *from a collagen coated surface was found by others to be much greater than 3.9 pN and on a fibronectin coated surface to be between 15-26 pN [51,52]. Therefore significant cell detachment may not be occurring under the conditions of this study. Catch bonds, which increase in strength with increasing shear stress, could be forming more frequently at the lower shear stress condition (0.24 Pa) leading to increased cell adhesion under these conditions [53,54].

MRSA exposure time affected the extent of infection under both static and flow conditions. For static experiments, endothelial binding sites may have been saturated, as there was no increase in the number of MRSA per HUVEC between 30 minutes and 60 minutes, and no significant change in the percent HUVEC infected was observed over the time course. This agrees with findings of Tompkins et al. [48] who saw saturation within 60 minutes for static adhesion using similar bacterial inoculum sizes. Under flow at 293s^-1^, a very different result was found. There was no change in the number of MRSA per HUVEC between 15 and 30 minutes, but there was a 260% increase between 30 and 60 minutes. The presence of flow appears to be exacerbating the endothelial response to infection at the one hour time point, perhaps leading to modulation of surface properties that increase bacterial uptake. Further studies are needed to investigate the mechanisms involved.

## Conclusions

This study demonstrated that MRSA adheres to endothelium in a shear dependent manner which may be affected by the non-uniform shear stress distribution over an undulating endothelial monolayer. MRSA adhered to and invaded HUVEC, with a large degree of heterogeneity. The time course of adhesion under flow conditions varied greatly as compared to static. Therefore, the mechanisms of adhesion under flow may differ from those seen under static conditions and thus warrant further investigation. During establishment of blood borne infections, bacteria contact endothelial cells throughout the vasculature. Our results suggest that at increasing time points, MRSA will adhere in increasing numbers under low flow, an effect not seen in static culture. Further, adhesion appears to be dependent on shear stress magnitude. The number of MRSA increased between the static and low flow conditions. A further increase in shear stress led to a decrease in MRSA numbers. Therefore, MRSA appear to adhere and invade endothelial cells preferentially in regions of low shear stress, such as those found in vascular branches and areas of curvature. Taken together, these findings suggest that shear stress and time each influence MRSA adhesion to, and internalization by, endothelial cells. While steady flow was assessed in this work, further studies investigating the role of flow pulsatility and recirculation should provide further insights into the role of fluid dynamics on bacterial adhesion in the vasculature.

## Competing interests

The authors declare that they have no competing interests.

## Authors' contributions

KDV carried out the cell-based studies under the direction of RDS and KDR. SSD and MMS performed the micro-PIV and CFD studies, respectively, under the direction of RMM. KDV, SSD, MMS, RDS and KDR drafted the manuscript. KDR and RMM supervised the project and participated in its design and data analysis. All authors read, edited and approved the final manuscript.
